# The anatomy of well-being: understanding its psychosocial and sociodemographic dimensions

**DOI:** 10.3389/fsoc.2025.1691938

**Published:** 2025-10-21

**Authors:** Carlos Barros, Mariana Malta Cruz, Margarida Gaspar de Matos

**Affiliations:** ^1^Research Centre for Psychological, Family and Social Well Being (CRC-W), Universidade Católica Portuguesa, Lisboa, Portugal; ^2^Research Centre for Communication and Culture (CECC), Universidade Católica Portuguesa, Lisboa, Portugal

**Keywords:** well-being, migrations, psychosocial factors, sociodemographic factors, integration, social determinants

## Abstract

Migration processes should be analyzed in terms of their psychosocial impact within a multisystemic context. This study aims to identify the psychosocial and sociodemographic factors that influence the well-being of Portuguese migrants, focusing on aspects such as educational qualifications, health, employment status, the length of time they have been outside Portugal and where they live. Participants are 395 Portuguese emigrants currently living abroad, aged between 20 and 78 (*M* = 37.60, *SD* = 8.65). The participants live in various geographical locations. About three-quarters (77%, *n* = 302) of respondents lived in European countries, particularly in urban areas (71%, *n* = 281), mainly women (78%*, n* = 308). Linear regression models used as independent variables gender, health, marital status, qualifications, professional status, residence, age and years since emigrating and as dependent variables the satisfaction with social ties, connection, cohesion, acculturation and adaptation. Data were collected through an online survey using non-probabilistic recruitment, and analyzed with ANOVA for mean comparisons and stepwise linear regression to identify the strongest predictors of well-being dimensions. The results suggest that interventions aimed at promoting migrants' well-being should integrate the social, physical, and mental dimensions of health, recognizing the importance of support networks, a sense of belonging, employment conditions, and community cohesion for adaptation and integration. The study emphasizes the importance of synergy between individual and contextual well-being in creating healthy spaces, populations and communities.

## 1 Introduction

Migration processes cannot be understood solely through individual or collective lenses. Instead, they should be analyzed in terms of their psychosocial impact, where relational dynamics are embedded in a multisystemic context. This perspective is essential to understanding how the migration project is experienced by individuals and groups (Bryceson, 2019; Julca, 2011).

Keyes and Shapiro (2004) describe well-being as an individual's perception of the quality of their relationships with others, particularly with their neighbors and the surrounding community. Although some authors offer a conceptualization of the term, there is no real consensus on its definition in scientific literature, making operationalizing and evaluating this construct more difficult. However, well-being has not only always been at the center of philosophical concerns, when analyzed, it becomes impossible not to address the subject of health (e.g. Larson, 1993).

The relationship between physical and psychological health is now an indisputable fact. Before World War II, the concept of well-being was based on the mere absence of disease. However, after 1948, the World Health Organization (WHO) proposed a more comprehensive definition, considering that well-being is not merely the absence of disease, but above all a comprehensive state of physical, mental and social health (Bobowik et al., 2015; Cooke et al., 2016).

Similarly, Keyes and Shapiro (2004) consider that the relationship between social issues and health—which leads to well-being—is particularly important, despite the fact scientific literature does not adequately address this relationship. The authors point out that well-being must be social and consists of several elements which, together, indicate whether and to what extent individuals function well in their social lives—for example, as neighbors, colleagues and citizens. According to the authors, subjective well-being should be conceptualized on a continuum defined by different levels of analysis, specifically by individual, interpersonal and societal aspects (Keyes and Ryff, 1998).

Recent literature demonstrates there has been an effort to bring the dimension of social health to the center of the discussion. Doyle and Link (2024) present a conceptualization of the dimension itself, covering a person's ability to form and maintain satisfactory relationships, adapt to social norms, contribute to the community and integrate socially, which can be seen simultaneously as a resource—facilitating and sustaining physical and mental health; and as a result—an indicator of overall well-being.

Complementarily, Fleuret and Atkinson (2007) propose a framework based on the concept of spaces of well-being, which identifies four types of spaces that support well-being: spaces of capacity, which facilitate self-realization; integrative spaces, which strengthen social networks and cohesion; spaces of safety, which reduce risks and increase the feeling of protection; and therapeutic spaces, which promote physical, mental and social healing, highlighting that well-being is socially and culturally constructed and depends on the opportunities and constraints of the place where people live, including their ability to participate, connect and feel safe.

From a sociological perspective on well-being, Larson (1993) states that this construct relates to two interrelated dimensions: social adjustment and social support. The first is closely related to individuals‘ satisfaction in interpersonal relationships, their perception of their individual performance in different social roles, and their environmental adjustment; social support, on the other hand, is related to the number and quality of the subject's social contacts, as well as their ability to relate to others. Keyes and Ryff (1998), later corroborated by Keyes and Shapiro (2004), consider that there are five dimensions related to well-being: *i)* social integration, which refers to the subjective assessment of the type and nature of the relationship that the subject establishes with the surrounding community. Social integration is closely associated with a sense of belonging to the world and to others; *ii)* social contribution, which relates to the subjective perception of a person's value in society and the active contributions they make or can make to the common good; *iii)* social coherence—consists of the subjective perception of the quality, organization and mastery of the social world, including a concern for knowledge of aspects of life and its existence; *iv)* social actualisation, which relates to the subjective assessment of individuals' potential and its expression in the world; and *v)* social acceptance, which relates to levels of trust in others and the feeling of acceptance in one or more social groups. Complementary evidence suggests that social belonging and cohesion are central predictors of psychological well-being among migrant and minority populations (Martinez-Donate et al., 2023). Similarly, studies on adolescent and family well-being highlight how migration challenges can exacerbate vulnerabilities (Bastia, 2014; Salas-Wright et al., 2022).

The different definitions/conceptualizations of this construct were organized by Cooke et al. (2016) into four theoretical approaches: *i)* hedonistic approaches, *ii)* eudaimonistic approaches, *iii)* approaches related to quality of life, and *iv)* approaches related to well-being. Despite the differences between each model, they all share common aspects of the concept: “These four categories of approaches to understanding well-being have substantial similarities, with the broadest commonality being each construct's foundational interest in the positive dimension of human experience and functioning” (Cooke et al., 2016, p. 5). Similarly, Zhang et al. (2024), confirms the relevance of this categorization, while advocating the inclusion of complementary dimensions, such as physical health and generic happiness, to more comprehensively capture the complexity of well-being in diverse cultural contexts. Furthermore, contemporary integrative perspectives also emphasize that the hedonic and eudaimonic dimensions should not be seen as mutually exclusive, but as interdependent components of human *flourishing* (Huta and Waterman, 2014; Ryff, 2018).

Focusing on the migratory context, Wessendorf and Phillimore (2019) provide insights into social well-being through the concept of social integration, a process in which newcomers adapt socially, economically, politically, and culturally, while long-settled populations also adjust, leading to shared relationships, values, and practices. Social integration thus refers to the set of relationships that migrants establish within the host country, whether with individuals of the same national/regional identity or with individuals, institutions, or associations in the destination country (Wessendorf and Phillimore, 2019).

However, it is still important to consider that this happens in a cultural framework. Culture consists of a kind of collective programming that makes members of one society different from members of another society (Matusitz and Musambira, 2013). In this sense, culture has a decisive impact on perceptions, attitudes and behaviors. Consequently, in order to understand integration, it is necessary to consider acculturation, which can emerge as a cause-and-effect variable originating from migration. Hajro et al. (2019) defines acculturation as the process of change that occurs in individuals' behavioral repertoire at the level of their value system and in their personal identity and social perception after migration. Some authors (Berry, 2005; Hajro et al., 2019) consider that the acculturation process is closely associated with an increased effort to achieve physical and psychological well-being, which can influence life satisfaction, mainly due to adaptation to an environment that is unfamiliar to individuals.

The literature suggests some factors that can enhance or hinder, and even serve as measures of well-being in the migration process. In this sense, (Berry 1997, 2005) refers to four strategies employed by migrants to deal with acculturation in their migration process: *i) assimilation*, when individuals strive to initiate and maintain interactions with the culture of the country of arrival; *ii) separation*, when they remain very attached to the cultural values of their country of origin and avoid social interactions in the country of arrival; *iii) marginalization*, when migrants show little interest in adapting to the culture of the host country, mainly due to the loss of cultural references from their country of origin, leading them to develop a trajectory of exclusion and discrimination in the country of arrival; and *iv) integration*, when individuals remain attached to the cultural values of their country of origin and, in addition, actively seek to relate to the cultural values of the country of arrival. According to Hajro et al. (2019), there is a relationship between acculturation processes, coping strategies and the successful sociocultural integration of migrants. Research in this field shows that separation and marginalization strategies are associated with low levels of life satisfaction and poor relations among the migrant population, compared to those who use assimilation and integration strategies.

To further situate this study within contemporary European debates, recent empirical evidence has been integrated. (Pollenne and Vargas-Silva 2024) analyzed well-being trajectories of migrants in the United Kingdom, showing that although levels of subjective well-being converge with the local population over time, initial disparities between migrant groups persist. Similarly, Brandt and Kaschowitz (2024) proposed a framework on care and well-being in Europe, highlighting that health systems, social support, and integration policies are key determinants of migrants' well-being. Canova et al. (2024) examined health status upon arrival, emphasizing that pre-, during, and post-migration experiences strongly influence physical and mental health outcomes. Finally, (Schäfer and Morillas 2025) investigated highly skilled migrants in Denmark and showed how professional and personal life intersect with social integration to shape well-being. Together, these studies demonstrate that European migration contexts have specific dynamics that help to understand the specificity (and transversality) of the Portuguese context.

A study of Portuguese migrants by Neto (2019) indicates that acculturation is easier for migrants who have been in the country for longer, pointing to the importance of the length of the migration project. Furthermore, the greater the integration in the destination country, the easier acculturation and adaptation. On the contrary, close ties with the country of origin/community of origin prove to be more difficult for acculturation and adaptation to the host country.

Furthermore, as predictors of well-being in the migration project, the following factors were identified: *i)* proficiency in the language of the destination country, *ii)* cultural identity proximity, and *iii)* defined acculturation strategies are positively related to the migration experience (Neto, 2002, 2006; Neto and Barros, 2007). Thus, some interdisciplinary authors refer to the importance of demographic variables (e.g. age, gender, level of education, marital status, employment status, area of residence) that should be considered for a more nuanced understanding of well-being and creation of social responses for these populations (Barros and Hanenberg, 2024; King, 2011). In particular, research has found that employment setting plays a complex role in the well-being of migrants. Stable employment is often considered a protective factor, but several studies have shown that it can also lead to dissatisfaction under certain conditions. Situations of overqualification, professional mismatch (discrepancies between migrants‘ qualifications and their roles) and work-related stress are common in European migration contexts and can have a negative impact on subjective well-being. For instance, Battisti et al. (2019) discovered that highly qualified migrants frequently encounter overqualification in European labor markets, a phenomenon linked to lower life satisfaction. (Fleischmann and Höhne 2013) also noted that structural job mismatch can hinder migrants' integration and subjective well-being. More recently, (Muñoz-Comet and Miyar-Busto 2025) and Kim (2024) have highlighted that even highly skilled migrants face barriers to recognition in the labor market, resulting in frustration and poorer psychosocial adaptation. These findings emphasize that employment can function as both a resource for economic stability and a potential source of dissatisfaction when expectations and qualifications are not recognized.

This study aims to identify the psychosocial and sociodemographic factors that influence the well-being of Portuguese migrants. In an exploratory approach and focusing on aspects such as educational qualifications, health, employment status, the length of time they have been outside Portugal and where they live, the research attempts to understand how these variables relate to satisfaction with social ties, connection, cohesion, adaptation and acculturation.

To this end, we intend to explore the following research questions: How do sociodemographic factors influence individuals' resources for social integration? How do contextual and personal factors shape well-being? What dimensions underpin sociocultural adaptation? How does the length of the migration process affect integration? Finally, we seek to understand the subjective determinants of social cohesion and connection in mobility.

## 2 Materials and methods

### 2.1 Participants

Participants are 395 Portuguese emigrants currently living abroad, aged between 20 and 78 (*M* = 37.60, *SD* = 8.65). The participants live in various geographical locations.

About three-quarters (77%, *n* = 302) of respondents lived in European countries, particularly in urban areas (71%, *n* = 281). Of all participants residing outside Europe (23%, *n* = 93), 42 (~10%) resided in North, Central, or South America, 24 (6%) resided in Asian countries, 21 (5%) had residence in Africa, and 6 migrants (~2%) lived in Australia/New Zealand. On average, participants have resided in the host country for 9.72 years (*SD* = 8.98).

A higher proportion of women (78%*, n* = 308) were observed, and more than half of these were married (*n* = 160) or in a common-law relationship (*n* = 52). Similarly, more than half of the male participants were married (*n* = 37) or in a common-law relationship (*n* = 23). Most participants had completed higher education (*n* = 295, 75%) and around 87% were employed.

A higher percentage of women (81%) have a higher education degree compared to men (55%). Furthermore, although men and women have similar employment rates (91% for men and 86% for women), unemployed men are retired (7%), while unemployed women are either unemployed (5%) or inactive (~ 6%).

On average, life satisfaction was high (*M* = 5.75, *SD* =0.93; *Mdn* = 6.00) with only a small proportion of migrants considering themselves unhealthy (11%), regardless of gender.

We observed that the educational qualifications of migrants increased substantially, from less than 50% of secondary school students in the 1980s to more than 80% in recent years. This trend is accompanied by a decrease in participants with secondary education or lower qualifications, possibly reflecting the development of the educational and social system. Table 1 contains sociodemographic data.

**Table 1:** Categorical sociodemographic characteristics (*N* = 395).

	***N***	**%**
**Gender**		
Male	85	21
Female	308	78
**Marital status**		
Single	97	24.6
Common-law marriage	75	19
Married	19	50
Widowed	1	0
Divorced—separated	2	6
**Qualifications**		
No educational certificate	13	3
Primary/basic education	3	0
Secondary education	49	12
Vocational training	3	8.9
Higher education (3 years)	131	33
Higher education (4+ years/Master's degree)	118	29
Doctorate	4	11
**Professional status**		
Employed	342	86.6
Student	12	3
Unemployed	17	4.3
Not in the labor force	18	4
Retired	6	1
**Residence**		
Rural	28	7.1
Suburban	86	21
Urban	281	71
**Geographical area**		
European area	303	76
Rest of the world	92	23
**Years of emigration**	**9**	**8**

### 2.2 Instruments

#### 2.2.1 Socio-cultural adaptation

Twelve items from the Brief Socio-cultural Adaptation Scale (BSAS; Demes and Geeraert, 2014) were adapted. These items were rated on a 7-point Likert scale ranging from 1 “strongly disagree” to 7 “strongly agree” [e.g., I am comfortable with the climate of my host country (temperature, rainfall, humidity)]. Likewise, in the sociocultural adaptation scale, the items chosen seem to reproduce, through the confirmatory factor analysis, the adjusted factorial solution proposed by the authors, revealing a good quality of fit [χ2 (51) = 158.09, *p* < 0.001, CFI = 0.91, TLI = 0.89, RMSEA = 0.08, 90% CI RMSEA (0.06, 0.09), SRMR = 0.06], and confirming that the original factor structure of the scale has not changed. Cronbach's alpha revealed good internal consistency (α = 0.80).

#### 2.2.2 Acculturation orientation

Six items (e.g., I have friends in my host country) were adapted from the Brief Acculturation Orientation Scale (BAOS; Demes and Geeraert, 2014). Migrants rated their agreement on a 7-point Likert scale ranging from “strongly disagree” to “strongly agree”. The confirmatory factor analysis revealed that, in the acculturation orientation scale, the items chosen seem to reproduce the adjusted factorial solution proposed by the authors, revealing a good quality of fit [χ2 (15) = 29.08, *p* < 0.001, CFI = 0.93, TLI = 0.85, RMSEA = 0.09, 90% CI RMSEA (0.06, 0.13), SRMR = 0.05] and confirming that the original factor structure of the scale has not changed. Cronbach's alpha revealed satisfactory internal consistency (α = 0.61).

#### 2.2.3 Social cohesion

We used five items adapted from Boreham et al. (2013) to measure social cohesion (e.g. people here are willing to help their neighbors). One item was reverse-coded (e.g. in general, people in this neighborhood do not get along well), so higher scores on the composite index reflect higher levels of social cohesion. The confirmatory factor model, adjusted to the sample, revealed a very good quality of fit [χ2 (4) = 10.57, *p* = 0.032, CFI = 0.99, TLI = 0.97, RMSEA = 0.07, 90% CI RMSEA (0.02, 0.12), SRMR = 0.03], confirming that the factorial structure of the scale proposed by the authors does not change. Internal consistency was adequate (α = 0.79).

#### 2.2.4 Perceived satisfaction with social connections

The social connections index included four items (e.g. I spend leisure time with my work colleagues) and was adapted from *the World Values Survey* (Inglehart and Wenzel, 2005). Responses were given on a 7-point Likert scale, ranging from ‘very dissatisfied' to ‘very satisfied'. The confirmatory factor analysis shows that the selected items seem to reproduce the adjusted factorial solution proposed by the authors, revealing a very good quality of fit [χ2 (1) = 1.71, *p* = 0.191, CFI = 1.00, TLI = 0.99, RMSEA = 0.04, 90% CI RMSEA (0.00.15), SRMR = 0.01] and confirming that the original factor structure of the scale has not changed. The internal consistency of the composite index was good (α = 0.85).

#### 2.2.5 Perceived health and life satisfaction

Migrants' perception of their health status was measured with an item in which participants were asked to rate their health status on a 5-point Likert scale, ranging from “poor” to “excellent”. Perceived life satisfaction was calculated using a composite index (α = 0.85 =0.74 in the present study) of 5 items adapted from Boreham et al. (2013). The items were measured on a 7-point Likert scale ranging from “very dissatisfied” to “very satisfied”, addressing different aspects of the migrant's life (e.g. their leisure opportunities). According to Diener et al. (1985), the alpha correlation coefficient is 0.87, which indicates adequate levels of internal consistency.

#### 2.2.6 Sociodemographic variables

The sociodemographic variables considered included age, gender and marital status (single, cohabiting, married, widowed or divorced) of the participants. Their educational qualifications (from primary to higher education) and professional status (student, employed, unemployed, inactive, retired) were also considered. Finally, participants were also asked to identify their host country and length of residence, as well as the rural, suburban or urban context in which they live.

The variables studied and their respective instruments are summarized in Table 2.

**Table 2:** Study variables and measures.

**Variable type**	**Variable**	**Instrument/source**	**Items**	**Scale**	**Example item**
Sociodemographic	Age	Self-report	1	Continuous	—
Sociodemographic	Gender	Self-report	1	Male/Female	—
Sociodemographic	Marital status	Self-report	1	Categories	Single, married, divorced, etc.
Sociodemographic	Education	Self-report	1	Categories	Secondary, Bachelor, Master, etc.
Sociodemographic	Professional status	Self-report	1	Categories	Employed, unemployed, student, etc.
Sociodemographic	Residence	Self-report	1	Categories	Rural, suburban, urban
Sociodemographic	Years emigrated	Self-report	1	Continuous	—
Psychosocial	Social ties satisfaction	World values survey (Inglehart and Wenzel, 2005)	4	Likert 1–7	I spend leisure time with my colleagues.
Psychosocial	Social connection	World values survey	4	Likert 1–7	I have strong social bonds in my community.
Psychosocial	Social cohesion	Boreham et al. (2013)	5	Likert 1–7	People here are willing to help their neighbors.
Psychosocial	Acculturation orientation	BAOS (Demes and Geeraert, 2014)	6	Likert 1–7	I have friends in my host country.
Psychosocial	Sociocultural adaptation	BSAS (Demes and Geeraert, 2014)	12	Likert 1–7	I am comfortable with the climate of my host country.
Psychosocial	Health perception	Self-report	1	Likert 1–5	How would you rate your health?
Psychosocial	Life satisfaction	Boreham et al. (2013)	5	Likert 1–7	How satisfied are you with your leisure opportunities?

### 2.3 Procedure

#### 2.3.1 Data collection

The sampling procedure was non-probabilistic, with emigrants recruited online. The requirements for participation were: *i)* to be an emigrant of Portuguese nationality in any country in the world; *ii)* to be over 18 years of age.

With the support of Portuguese communities abroad, parish councils/local authorities in Portugal, the media, emigrant associations, as well as the creation of a website and social media pages for the research project, the study was publicized to recruit participants. Migrants were invited to participate in the study through materials disseminated through these channels and informed about the objectives of the study.

The anonymity of participants' personal and digital data was guaranteed, and only questionnaires for which online consent was approved by participants were included. Survey data were collected online through the *Qualtrics* platform (Provo, UT) during 2019.

The study followed the codes of ethics of the American Sociological Association (2018) and the American Psychological Association (2018) in accordance with the Helsinki Declaration.

#### 2.3.2.Data analysis

Data were analyzed using IBM SPSS Statistics, version 30 (IBM Corp., Armonk, NY, USA). Simple basic descriptives were carried out, as shown in Table 3.

**Table 3:** Means and standard deviations.

	***M***	***SD***
Satisfaction with social ties	5.75	0.93
Connection	4.47	1.36
Cohesion	5.01	0.64
Acculturation	5.24	0.84
Adaptation	5.18	0.88

ANOVA was used, with Tamhane's T2 post-hoc test applied when variances were unequal (heteroscedasticity), and Welch's ANOVA reported when assumptions were violated. Effect sizes (η^2^ or ω^2^) are presented alongside *p*-values. The Stepwise method was used as an exploratory strategy to select independent variables and identify the strongest predictors. Categorical predictors were entered using dummy coding The independent variables used were gender, health, marital status, qualifications, professional status, residence, age and years since emigrating. The dependent variables used were, in order, satisfaction with social ties, connection, cohesion, acculturation and adaptation.

All instruments were translated into European Portuguese with harmonization and pilot testing. Reversed items were recoded. Cross-group comparability was not tested and is noted as a limitation.

## 3 Results

### 3.1 Satisfaction (with social ties)

Satisfaction with social ties was compared by category for each of the independent variable.

Regarding qualifications, ANOVA identified significant differences (*F*= 7.393, *p* < 0.001). *Tamhane's* test showed differences between secondary qualification and master's degree (*p* < 0.05), secondary qualification and doctorate (*p* < 0.001), professional qualification and master's degree (*p* < 0.05), professional qualification and doctorate (*p* < 0.001) and bachelor's degree and doctorate (*p* < 0.05).

ANOVA also identified significant differences (*F* = 9.994, *p* < 0.001) regarding health. The *Tamhane*'s test detected differences between fair and good (*p* < 0.05) and fair and excellent (*p* < 0.001).

Regarding professional status, ANOVA identified significant differences (*F* = 3.963, *p* < 0.05) between employed and unemployed (*p* < 0.05). It should be noted that the degree of satisfaction is higher among employed individuals, decreasing among students and even more so among the unemployed, corroborating the conclusion of an inverse association between satisfaction and professional status.

The regression model with these three independent variables (qualifications, health, professional situation and region) was found globally adequate (*F* = 21.556, *p* < 0.001), explaining 13,8% of the variance. Qualifications and health both positively influenced satisfaction (β_1_ = 0.165 and β_4_ = 0.265). Professional status, on the other hand, although significant (*p* < 0.05), negatively influenced satisfaction (β_3_ = −0.147). In summary, more qualifications and better health lead to greater satisfaction; however, better professional status tends to decrease satisfaction. Given that the variance explained by the models ranges only between 4–14%, the results should be seen as exploratory rather than confirmatory.

The results of the regression model for satisfaction with social ties can be consulted in Table 4.

**Table 4:** Regression model for satisfaction with social ties.

**Model**	**Unstandardized coefficients**		**Standardized coefficients**	***t***	**Sign**.

	**B**	**Standard error**	**Beta**		
(Constant)	3.659	0.344		10.634	< 0.001
Qualifications	0.165	0.033	0.244	4.977	< 0.001
Health	0.265	0.069	0.187	3.855	< 0.001
Sit_Prof	−0.147	0.052	−0.134	−2.803	0.005

a. Dependent variable: satisfaction.

### 3.2 Connection

The ANOVA identified (*F* = 6.370, *p* < 0.001) significant differences in the connection and health, namely between fair and excellent and good and excellent (*p* < 0.05). As for residence, ANOVA results showed significant difference (*F* = 4.686, *p* < 0.05) between suburban and urban areas (*p* < 0.05). Specifically, the average connection increases from rural to urban regions.

The regression model, with the three selected independent variables, is globally an adequate model (*F* = 10.342, *p* < 0.001), explaining 6.8 % of the variance. Health (β_1_ = 0.403), residence (β_2_ = 0.336) and years as an emigrant (β_3_ = 0.020) positively influence connection. In summary, better health, more years as an emigrant, and residing in suburban and urban areas lead to greater connection.

The analysis of the connection model can be seen in Table 5.

**Table 5:** Regression model for connection.

**Model**		**Unstandardized coefficients**		**Standardized coefficients**	***t***	**Sig**.
		**B**	**Standard error**	**Beta**		
3	(Constant)	1.715	0.518		3.314	0.001
	Health	0.403	0.101	0.196	3.984	< 0.001
	Residential	0.336	0.110	0.151	3.054	0.002
	Years_emigrated	0.020	0.008	0.127	2.577	0.010

a. Dependent variable: connection.

### 3.3 Cohesion

The result of the ANOVA showed a significant difference in cohesion (*F*= 8.279, *p* < 0.001) between fair and good health (*p* < 0.05), as well as between fair and excellent (*p* < 0.001).

The regression model proved to be acceptable (*F* = 18.680, *p* < 0.001), explaining 4.4 % of the variance. Health was found positively influencing cohesion (β = 0.207, *p* < 0.001), showing that the better the health, the greater the cohesion.

The analysis of the cohesion model can be seen in Table 6.

**Table 6:** Regression model for cohesion.

**Model**		**Unstandardized coefficients**		**Standardized coefficients**	***t***	**Sig**.
		**B**	**Standard error**	**Beta**		
1	(Constant)	4.142	0.202		20.470	< 0.001
	Health	0.207	0.048	0.215	4.322	< 0.001

a. Dependent variable: cohesion.

### 3.4 Acculturation

ANOVA did not identify differences in acculturation means by health status (*F*= 0.168, *p* =0.918).

The regression model with the number of years emigrated to explain acculturation was acceptable (*F* = 4.057, *p* < 0.05). We found that the number of years emigrated directly influences acculturation (β = 0.01), albeit with a very small weight.

The analysis of the acculturation model is presented in Table 7.

**Table 7:** Regression model for acculturation.

**Model**		**Unstandardised coefficients**		**Standardized coefficients**	***t***	**Sig**.
		**B**	**Standard error**	**Beta**		
1	(Constant)	5.148	0.063		81.163	< 0.001
	Years_emigrated	0.010	0.005	0.102	2.014	0.045

a. Dependent variable: acculturation.

### 3.5 Adaptation

ANOVA revealed that the adaptation means are not all equal in the health categories (*F* = 10.799, *p* < 0.001), specifically there was a significant difference between poor and excellent (*p* < 0.05).

The regression model using health to explain adaptation proved to be globally acceptable (*F* = 10.799, *p* < 0.001). The relationship between health and adaptation is direct (β = 0.220, *p* < 0.05).

The analysis of the adaptation model is presented in Table 8.

**Table 8:** Regression model for adaptation.

**Model**		**Unstandardized coefficients**		**Standardized coefficients**	***t***	**Sig**.
		**B**	**Standard error**	**Beta**		
1	(Constant)	4.267	0.283		15.096	< 0.001
	Health	0.220	0.067	0.166	3.286	0.001

a. Dependent variable: adaptation.

## 4 Discussion

Data highlights the importance of interactions between psychosocial and sociodemographic factors in understanding the well-being of the migratory experience among Portuguese individuals. This finding offers a relevant contribution to understanding the processes of integration, adaptation, and social cohesion in contexts of international mobility.

Beginning with health, which is perceived as cross-cutting and impacting various dimensions, such as satisfaction with social ties, sense of connection, cohesion and adaptation. We highlight the integrated view of perceived health (essential to well-being), in line with more systemic perspectives (Cooke et al., 2016; Keyes and Ryff, 1998; Keyes and Shapiro, 2004; Zhang et al., 2024). In this context, health, usually seen as tied to social integration (Larson, 1993), is again a key part of migration, since people in better health tend to have stronger social ties, community cohesion, and the ability to adapt.

Health is central to the explanation of sociocultural adaptation. Individuals in good health seem to demonstrate greater adjustment to their new context. This result reinforces the need for public policies and community actions that ensure equitable access to health services for migrants and training for professionals to deal with this flow (Barros and Hanenberg, 2024). In addition to individual health, social factors such as access to healthcare, experiences of discrimination and support from transnational families also influence well-being.

Social cohesion was explained exclusively by perceived health. Physical and emotional well-being emerge as catalysts for interpersonal trust and participation in community dynamics. In contrast, states of fragility tend to generate social isolation and feelings of exclusion (Keyes and Shapiro, 2004). Moreover, the concept of social health proposed by Doyle and Link (2024) reinforces this view, defining it as the adequate quantity and quality of relationships necessary to satisfy the human need for meaningful connection, arguing that this is a health outcome in itself and not just a determinant of other dimensions.

The length of residence in the host country proved to be a factor that facilitated the formation of social ties. As pointed out by previous studies, the duration of migration contributes to more stable acculturation processes and stronger interpersonal relationships (Neto, 2019). However, although there is an association between emigration duration and acculturation, the explanatory impact of this factor was modest. This suggests that the simple passing of time is not sufficient to guarantee profound changes in identity or behavioral patterns. Elements such as attitude toward the local culture, level of social exposure and support received must also be considered (Berry, 2005). Furthermore, according to Fleuret and Atkinson's (2007), well-being should be understood within a multidimensional framework that integrates social, economic, environmental and cultural factors, recognizing that health is built through interactions between individuals and the spatial and social contexts in which they live.

The relationship between academic qualifications and social ties shows that higher levels of education are associated with greater satisfaction with social ties. A possible explanation could be benefiting from interpersonal, cultural and linguistic skills that facilitate integration, which come from longer training, people tend to develop more extensive and diverse support networks (Berry, 1997).

Living in urban areas has been associated with a greater perception of belonging and the formation of stronger social networks. The concentration of resources and opportunities to interact in metropolitan areas seems to favor social integration (Wessendorf and Phillimore, 2019). This may also be due to the existence of greater intercultural diversity in urban spaces, as well as the greater presence of services, community groups (e.g. the diaspora), and easier access to employment.

The professional situation had a negative impact on social satisfaction. This effect may arise, in part, from tensions between work demands and the time available to invest in personal relationships; and, on the other hand, could indicate job mismatch and overqualification, as discussed by Battisti et al. (2019) and (Fleischmann and Höhne 2013). These dynamics help to explain why employment can become a source of frustration and reduced well-being among migrants, despite generally being protective.

## 5 Conclusions

This study invites us to reflect on the importance of considering well-being as a systemic approach, includes psychosocial and demographic dimensions. The findings of this study indicate that perceived health is the primary driver of migratory well-being. The analysis of the data collected from the sample suggests a positive correlation between health status and satisfaction with social ties, connection, cohesion, and sociocultural adaptation. A positive correlation has been observed between academic qualifications and satisfaction with social ties. Conversely, professional status has been found to have an unexpected negative effect, which may be attributable to overqualification and a lack of time for relationships. This phenomenon highlights the ongoing need for work-life balance which in determinant in the well-being. Furthermore, it was found that living in urban areas increases perspectives of social connection, and that longer periods of emigration strengthen connection and, to a modest extent, acculturation. Thus, it is essential to understand that connection to people and services can greatly increase well-being, which should not be viewed solely from an individual perspective, but rather through a systemic community lens.

The results suggest that interventions aimed at promoting migrants' well-being should integrate the social, physical, and mental dimensions of health, recognizing the importance of support networks, a sense of belonging, and community cohesion for adaptation and integration (Doyle and Link, 2024). This perspective is in line with the guidelines of the World Health Organization, which advocates the operationalisation of the social dimension of well-being as an integral part of health policies, promoting social cohesion, inclusion and community participation as key determinants. In this way, strategies that strengthen spaces for well-being (e.g. capacity, integrative, safety, and therapeutic) can create conditions for the development of meaningful and sustainable relationships (Fleuret and Atkinson, 2007). It is essential to rethink the approaches taken so far and make them more eclectic, valuing this dimension of social well-being in an applied way, through measures and metrics.

### 5.1 Pratice implications and limitations

Several practical implications can be identified, namely the development of community programmes that promote intercultural contact and reduce isolation, taking advantage of existing networks such as migrant and diaspora associations, also the transnational network family (Barros and Hanenberg, 2024; Jetten et al., 2017; Guerra and Barros, 2025), the implementation of health policies that consider social health as an indicator of well-being, monitoring not only physical and mental conditions, but also the quality and quantity of social relationships (Doyle and Link, 2024), the creation of spaces of well-being (capacity, integrative, safe and therapeutic) that respond to the specific needs of migrant populations, including cultural and linguistic aspects (Fleuret and Atkinson, 2007), the training of health, education and social services professionals to recognize signs of isolation and barriers to integration, intervening early, among others. These ideas can help mitigate the risks of isolation and improve migrants' adaptation.

In order to ensure good practices and their transfer, it is also necessary to consider the limitations of the present study, as well as suggestions for future research: the absence of statistically significant effects associated with gender or marital status may reflect a homogenisation of migratory experiences among traditionally differentiated groups, although this trend may also be conditioned by the composition of the sample analyzed. A further limitation is related to the use of stepwise regression. This method was chosen because it allows an exploratory identification of the most relevant factors, but it is known to have weaknesses, such as producing results that may not always generalisable. Future studies could adopt more robust methods, such as hierarchical regression or structural equation modeling, to strengthen the conclusions. Future research could use more robust methods, like hierarchical regression or structural equation modeling, to strengthen the conclusions. A further limitation is that we did not test whether the measures work the same way across groups (e.g., gender, host country). Intersectional analyses are also necessary to better capture complexity of realities. As the study relies exclusively on self-reported measures, potential biases such as social desirability and subjectivity must be considered. Furthermore, future studies may show the need for a sample that encompasses more profiles especially in terms of age, different educational qualifications, socioeconomic status, or region of residence, limiting the generalization of the results.

Finally, we would like to highlight some practical implications for interventions, such as the operationalization of the social dimension of health, including access to care, community belonging programmes and culturally and linguistically sensitive ‘well-being spaces', as well as attention to professional mismatch and working conditions.

## Data Availability

The datasets generated during the current study are not publicly available since they constitute an excerpt of research in a Doctoral study (Doctoral Grant from Fundação para a Ciência e Tecnologia, under reference PD/BD/128345/2017), with ethical protection for the participants. Still, general data are available from the corresponding author upon reasonable request. Research data is available in Portuguese.
