# Supplementary material for: The anatomy of well-being: understanding its psychosocial and sociodemographic dimensions
Source: Front Sociol. 2025 Oct 21;10:1691938. doi: 10.3389/fsoc.2025.1691938 (PMC12582946; doi:10.3389/fsoc.2025.1691938)
Supplement: Supplementary file 1 [file Supplementary_file_1.pdf]

Supplements to the manuscript

Appendix 1 – Visual representation of the Confirmatory Factor Analysis of the *Brief Sociocultural Adaptation Scale (BSAS)*

X^2 (51) = 158.093, $p < .001$, CFI = .91, TLI = .89, RMSEA = .08, 90% IC RMSEA [.06, .09], SRMR = .06

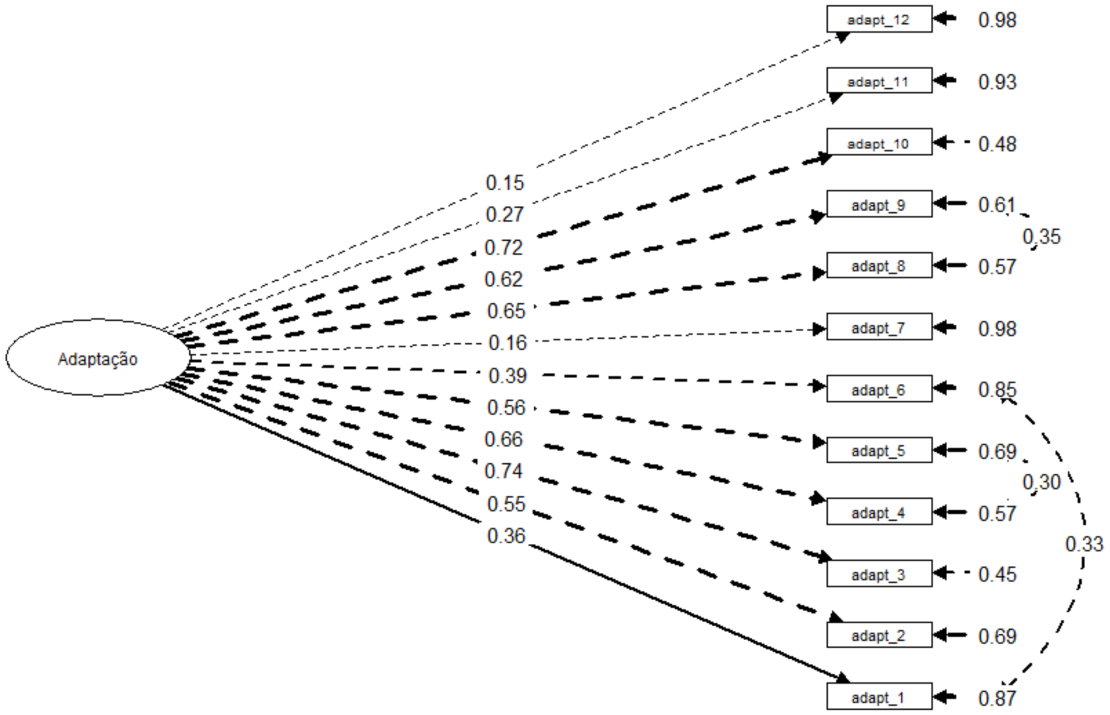

Appendix 2 – Visual representation of the Confirmatory Factor Analysis of the *Brief Acculturation Orientation Scale (BAOS)*

X^2 (15) = 29.083, $p < .001$, CFI = .93, TLI = .85, RMSEA = .09, 90% IC RMSEA [.06, .13], SRMR = .05

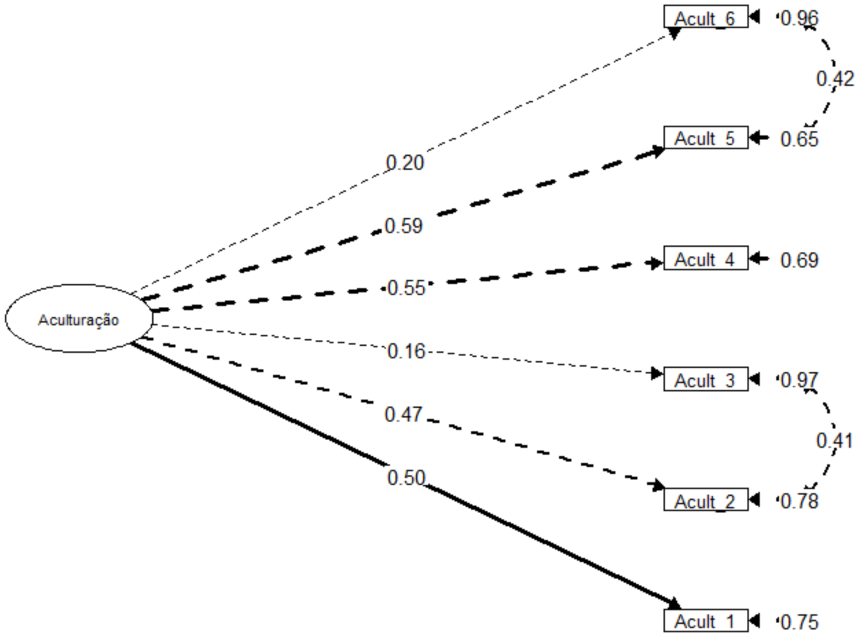

Appendix 3 – Visual representation of the Confirmatory Factor Analysis of Social Cohesion

X^2 (4) = 10.573, p = .032, CFI = .99, TLI = .97, RMSEA = .07, 90% IC RMSEA [.02, .12], SRMR = .03

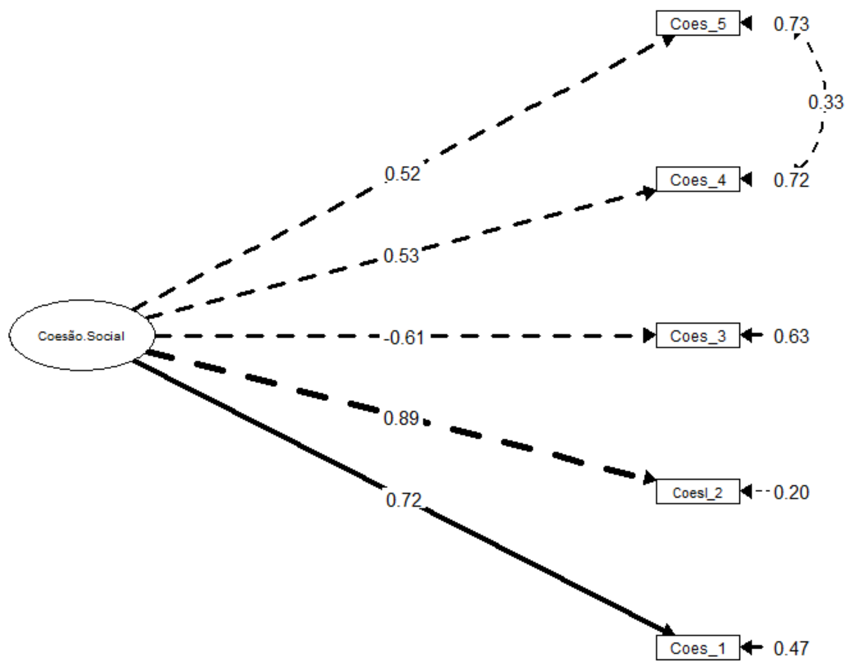

Appendix 4 – Visual representation of the Confirmatory Factor Analysis of Social Connection

X^2 (1) = 1.713, p = .191, CFI = 1.00, TLI = .99, RMSEA = .04, 90% IC RMSEA [.00 .15], SRMR = .01

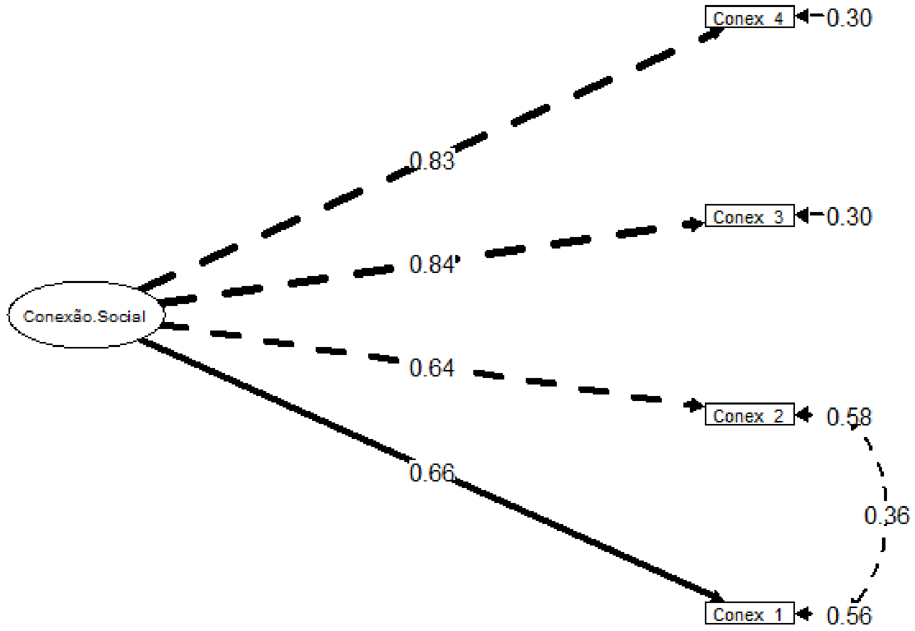

Conex_4
0.30
0.83
Conex_3
0.30
0.84
Conexão.Social
0.64
Conex_2
0.58
0.66
0.36
Conex_1
0.56
